# Sodium Butyrate Abrogates the Growth and Pathogenesis of *Mycobacterium bovis* via Regulation of Cathelicidin (LL37) Expression and NF-κB Signaling

**DOI:** 10.3389/fmicb.2020.00433

**Published:** 2020-03-19

**Authors:** Kai Zhang, Tariq Hussain, Jie Wang, Mengying Li, Wenjia Wang, Xiaojing Ma, Yi Liao, Jiao Yao, Yinjuan Song, Zhengmin Liang, Xiangmei Zhou, Lihua Xu

**Affiliations:** ^1^School of Agriculture, Ningxia University, Yinchuan, China; ^2^Key Laboratory of Animal Epidemiology and Zoonosis, Ministry of Agriculture, National Animal Transmissible Spongiform Encephalopathy Laboratory, College of Veterinary Medicine, China Agricultural University, Beijing, China; ^3^College of Veterinary Sciences, The University of Agriculture Peshawar, Peshawar, Pakistan; ^4^Institute of Laboratory Animal Sciences, Chinese Academy of Medical Sciences, Comparative Medicine Center, Peking Union Medical College, Beijing, China

**Keywords:** *Mycobacterium bovis*, sodium butyrate, histone deacetylation, Nuclear factor-κB, cathelicidin

## Abstract

*Mycobacterium bovis* is the causative agent of bovine tuberculosis, has been identified a serious threat to human population. It has been found that sodium butyrate (NaB), the inhibitor of histone deacetylase, can promote the expression of cathelicidin (LL37) and help the body to resist a variety of injuries. In the current study, we investigate the therapeutic effect of NaB on the regulation of host defense mechanism against *M. bovis* infection. We found an increased expression of LL37 in *M. bovis* infected THP-1 cells after NaB treatment. In contrast, NaB treatment significantly down-regulated the expression of Class I HDAC in THP-1 cells infected with *M. bovis*. Additionally, NaB reduced the expression of phosphorylated P65 (p-P65) and p-IκBα, indicating the inhibition of nuclear factor-κB (NF-κB) signaling. Furthermore, we found that NaB treatment reduced the production of inflammatory cytokines (IL-1β, TNF-α, and IL-10) and a key anti-apoptotic marker protein Bcl-2 in THP-1 cell infected with *M. bovis*. Notably, mice showed high resistance to *M. bovis* infection after NaB treatment. The reduction of viable *M. bovis* bacilli indicates that NaB-induced inhibition of *M. bovis* infection mediated by upregulation of LL37 and inhibition of NF-κB signaling pathway. These observations illustrate that NaB mediate protective immune responses against *M. bovis* infection. Overall, these results suggest that NaB can be exploited as a therapeutic strategy for the control of *M. bovis* in animals and human beings.

## Introduction

Tuberculosis is one of the deadliest infectious disease of human beings distributed worldwide, caused by *Mycobacterium tuberculosis*. The causative agent of bovine tuberculosis, *Mycobacterium bovis* also consider a serious threat to human population, which make this bacterium an important zoonotic specie of Mycobacterium complex (MTBC) (de la Rua-Domenech, 2006). In 2017, WHO report an estimated 10 million new cases of tuberculosis were recorded which caused 1.6 million deaths, a serious threat to the safety of human health and the livestock products processing industries (World Health Organization [WHO], 2018). As an intracellular bacteria, *M. bovis* evades multiple immune mechanisms of the host macrophages (Ernst, 2012). For example, *M. bovis* subvert the antibacterial abilities of infected macrophages by promoting the signaling pathways for the secretion of anti-inflammatory cytokines (Liu et al., 2017). Therefore, a better understanding of the interaction between *M. bovis* and host macrophages will help in the development of new strategies for the prevention and control of tuberculosis.

It has been reported that histone acetylation is regulated by histone deacetylase (HDAC) and histone acetyltransferase (HAT). HDAC can inhibit histone acetylation, while HAT counter act this effect (Shahbazian and Grunstein, 2007). By regulating the acetylation of histones, HDAC can regulate the expression of various gene and key biological process, including gene responsible for the production of inflammatory cytokines and its signaling pathways (Zhang et al., 2015). It has been reported that matrix metalloproteinase (MMP)-1 and -3 expression were regulated by HDAC in *M. tuberculosis* infection (Moores et al., 2017). In addition, HDAC1 can regulate the expression of iNOS and many cytokines (Calegari-Silva et al., 2018). HDAC3 and HDAC2 has been confirmed as a target of nuclear factor-κB (NF-κB)-mediated inflammation (Leus et al., 2018). It suggests that Class I HDAC might be crucial in the pathogenesis of *M. bovis*. In addition, emerging studies demonstrated the role of histone deacetylase inhibitors (HDACi) in the regulation of HDAC. Recently, numerous HDACi have been found to have anti-cancer, anti-epilepsy, regulating inflammatory response and other therapeutic effects (Suliman et al., 2012).

It is known that sodium butyrate (NaB) is a short chain fatty acid salt, secreted by butyrate-producing bacteria in the intestinal tract of the body (Trachsel et al., 2018). It is competitively compatible with the zinc regions of Class I and Class II HDAC, thereby inhibiting the activity of most Class I and Class II HDAC (Schwarz et al., 2017). Similar to HDACi, NaB also affects the transcriptional function of cellular gene and regulate various functions of the cell. Growing studies demonstrated that NaB can promote the expression of cathelicidin (LL37), one important antibacterial peptide (Liu et al., 2013). LL37 is mainly released by activated macrophages, monocytes, neutrophils and epithelial cells. In addition, it has been reported that LL37 is not only function as anti-microbial, anti-toxic activity or involve in immune regulation, but also participates in wound healing, and neovascularization. Previous studies reported that LL37 can resist the pathogenesis of *M. tuberculosis*, through direct killing of *M. tuberculosis* bacilli as well as regulating host cell immune responses such as the induction of autophagy (Rekha et al., 2015). In addition, recent studies found that NaB can promote tumor cell apoptosis in cancer (Pant et al., 2017).

Therefore, in the current study we hypothesized to investigate the effect of NaB on the host-immune responses during *M. bovis* infection. Furthermore, the aim of our study was to determine the potential role of NaB on the expression of LL37, and the regulation of NF-κB signaling pathway in *M. bovis*-infected macrophages. In addition, we sought to observe the therapeutic effect of NaB on C57BL/6 mice infected with *M. bovis*. Notably, we found that NaB efficiently increased the expression of LL37 in *M. bovis* infected macrophages. In addition, NaB significantly reduced the intracellular growth of *M. bovis* in macrophages. Furthermore, NaB treatment significantly reduced the pathogenesis of *M. bovis* in mice. These findings illustrate that NaB can be used as a potential new therapeutic candidate for the treatment of bovine tuberculosis.

## Materials and Methods

### Cell Culture

THP-1 cells were obtained from the Cell Culture Center, Xiehe Medical University (Beijing, China). The cells were cultured in RPMI 1640 (HyClone, Logan, UT, United States) medium supplemented with 10% FBS (Gibco, Grand Island, NY, United States), 100 μg/ml streptomycin and 100 U/ml penicillin (Gibco) at 37°C with 5% CO_2_ incubator. The cells were transferred to 6 and 12 wells culture plats in RPMI 1640 medium in the presence of 10 ng/ml phorbol 12-myristate 13-acetate (PMA, Sigma-Aldrich, P8139) for 48 h to differentiate into macrophage-like cells before further experiments.

### Bacterial Culture and Infection

Virulent *M. bovis* Beijing strain C68004 was provided by the China Institute of Veterinary Drug Control (CVCC, China). *M. bovis* was cultured in 7H9 Middlebrook medium (Difco) supplemented with 10% albumin-dextrose-catalase (ADC), 2 mg/L sodium pyruvate and 0.05% Tween-80 at 37°C in biosafety level 3 (BSL3) facility. *M. bovis* growth in broth medium was determined by calculating the OD value of the culture at 600 nm as described in our previous study (Wang et al., 2018). THP-1 cells were cultured in 6-well plates (1 × 10^6^ cells/well) for 48 h in the presence of PMA (10 ng/ml) then infected with *M. bovis* at multiplicity of infection (MOI) 10:1 for 3 h. After, 3 h of incubation cells were washed with sterilized phosphate-buffered saline (PBS) thrice to remove extracellular bacteria.

### Cell Viability Assay

The viability of THP-1 cells were determined by using MTS Kit according to the manufacturer’s instructions. THP-1 cell (1 × 10^4^ cells in each well) were cultured in 96-well plates in the presence of 10 ng/ml PMA in RMPI 1640 cell culture medium for 48 h. After incubation, THP-1 cells were washed with warm PBS thrice and treated with various concentration of NaB (0, 0.05, 0.25, 0.5, 1.0, 5.0, 10.0, 25.0, and 50.0 mM) in RPMI-5% FBS at 37°C for 24 h in CO_2_ incubator. Next, the cells were washed with PBS, then added 10 μl MTS solution to each well and incubated at 37°C for 4 h. After incubation the absorbance was measured at wavelength of 450 nm with a microplate reader (Thermo Scientific Multiskan FC, United States).

### Colony-Forming Units Assay

To assess total viable bacterial count, THP-1 cell (2 × 10^5^cells in each well) were cultured in 12-well plates in the presence of 10 ng/ml PMA in cell culture medium for 48 h followed by infection with *M. bovis* (MOI 10:1) for 3 h before the cells were washed to remove extracellular bacteria. After washing cells were treated with NaB 0.5 and 1 mM in RPMI-5% FBS at 37°C for 24 h. Thereafter, cells were incubated for the indicated time periods and lysed with 0.2% Triton X-100. For animal experiment lung and spleen tissues were collected soon after sacrificing at various time points to assess the effect of NaB on the proliferation of *M. bovis* in infected mice. The tissue samples were homogenized in PBS in a tissue homogenizer apparatus. For enumeration of *M. bovis*, colony-forming unit (CFU) assay was performed by making 10-fold serial dilutions for all tissue and cell samples in sterilized PBS. Equal volume from each dilution was inoculated in triplicate on Middlebrook 7H11 agar plates supplemented with 10% ADC, 0.05% Tween-80 and 2 mg/L sodium pyruvate at 37°C. Colonies of *M. bovis* were counted in each culture plate after 2–3 weeks of incubation.

### Quantitative Real-Time PCR

Total RNA from cells were extracted by using Trizol Reagent (Invitrogen), and the concentration and integrity of RNA was detected by using NanoDrop 2000 spectrophotometer (Thermo Scientific, Waltham, MA, United States). Reverse transcription of 100 ng RNA was performed by the RevertAid First Strand cDNA Synthesis Kit (Fermentas, Glen Burnie, MD, United States) according to the manufacturer’s instructions, and the integrity and concentration of cDNA were detected by using NanoDrop 2000 spectrophotometer. AceQ qRT-PCR SYBR Green Master Mix kit (Vazyme Biotech, Nanjing, China) was used for the amplification of mRNA’s gene, by using 700 Fast Real-Time PCR Systems (ViiA7 Real-time PCR, ABI). The primers used for qRT-PCR are as follows: *CAMP*, CAGGACGACAC AGCAGTCAC (Forward) and CTGGGTACAAGA TTCCGCAAA (Reverse); GADPH, GGAGCGAGATCCCTCCAAAAT (Forward) and GGCTGTTGTCATACTTCTCATGG (Reverse). The relative expression levels of mRNAs were determined by comparative Ct (2^–△△Ct^) method (Liao et al., 2019).

### Western Blot Assay

Total protein was collected from cells by using RIPA Lysis Buffer (Beyotime Ltd., Beijing, China). Equal amount of protein was separated by SDS-PAGE and then transferred onto PVDF membranes (Millipore Corporation, Billerica, MA, United States). After blocking with 5% skim milk in TBST buffer the membrane was probed for overnight at 4°C with primary antibodies for target proteins such as rabbit polyclonal anti-cathelicidin (LL37) antibody (ab180760) (Abcam, Cambridge, United Kingdom), rabbit polyclonal anti-Tublin-α, rabbit polyclonal anti-GAPDH antibody (10494-1-AP), rabbit polyclonal anti-IκBα antibody (10268-1-AP) (Proteintech, Wuhan, China), rabbit polyclonal anti-P65; RELA rabbit polyclonal antibody (10745-1-AP), rabbit polyclonal anti-HDAC3 antibody (10255-1-AP), rabbit polyclonal anti-Bcl2 antibody (12789-1-AP), rabbit polyclonal anti-Bax antibody (50599-2-lg) (Proteintech, Wuhan, China), rabbit monoclonal anti-phospho-NF-κB P65 (Ser536) (93H1) antibody (3033), mouse monoclonal anti-phospho-IκBα (ser32/36) (5A5) antibody (9246), rabbit monoclonal anti-HDAC1 (D5C6U) antibody (34589), rabbit monoclonal anti-HDAC2 (D6S5P) antibody (57156) (Cell Signaling Technology, Boston, MA, United States). After incubation the membranes were washed with TBST for 10–15 min followed by incubation with HRP-labeled secondary antibody (Proteintech, Wuhan, China) at 37°C for 1 h. The bands were developed with ECL substrate and visualized by using Bio-Rad Imaging System by applying ECL substrate as developer. ImageJ software (National Institute of Health, Bethesda, MD, United States) was used for the analysis of WB bands.

### ELISA for Cytokines

The level of IL-1β, IL-10, and TNF-α in cells culture supernatants were assessed by using ELISA technique. Briefly, standards, and samples were prepared according to the manufacturer’s protocols (Neobioscience, Shenzhen, China). Standards and samples (100 μl each) were added into appropriate wells of 96-well ELISA plates. After incubation, the plates were washed with a washing buffer followed by adding HRP-conjugated antibodies into each well for 60 min at 37°C. After that, added TMB substrate for 15 min at room temperature in dark followed by adding, stop solution into each well to stop the reactions. The OD values were measured at 450 nm by using an ELISA Plate Reader (Thermo Scientific Multiskan FC, United States). A standard curve was obtained by using twofold dilutions for calculating the concentration of cytokines.

### Mice Model of *M. bovis* Infection

C57BL/6 mice were obtained from SPF Biotechnology (Beijing, China) and were kept in cages under BSL3 laboratory facilities of China Agricultural University. Mice were infected with 200 CFU of virulent *M. bovis* via intranasal route (i.n.). The experimental groups of C57BL/6 mice were divided equally into the following three groups: (1) a vehicle control group: uninfected, only treated with PBS via intraperitoneal (i.p.) route with 50 μl PBS repeated after every 2 days; (2) a vehicle + *M. bovis* infected group: treated with 50 μl PBS via i.p. every 2 days post-infection with *M. bovis*; (3). *M. bovis* + 500 mg/kg NaB treated group: in which animals were treated with 500 mg/kg NaB via i.p. route every 2 days post-infection with *M. bovis*. Mice were sacrificed at 3rd, 5th, and 7th week post-infection and blood serum samples, lung, spleen, and liver tissues were harvested aseptically as soon as possible for further analysis.

### Histopathological Examination

Histopathological examinations were conducted to determine the effect of NaB treatment on various organs of mice infected with *M. bovis*. For evaluation of gross pathology, lung and spleen of experimental mice were weighed and clear images were captured at different time points of infection. For histological study, tissues were fixed in a 10% formaldehyde solution, embedded in paraffin, and cut into sections using a microtome. After mounting on microscopic glass slides, tissues were deparaffinized, and stained with: hematoxylin and eosin (H&E) for detail observation of lesions or stained with Ziehl–Neelsen (ZN) for visualizing the acid-fast *M. bovis* bacilli. Sections stained with H&E or ZN were observed under low and high magnification of microscope fitted with camera. Lung injuries were estimated by calculating the percentage of the area occupied by lesions in the total lung area using an Image-Pro macro software.

### Ethics Statement

All animal experiments were conducted under the protocols and procedures of the Chinese Regulations of Laboratory Animals—The Guidelines for the Care of Laboratory Animals (Ministry of Science and Technology of the People’s Republic of China) and Laboratory Animal Requirements of Environment and Housing Facilities (GB 14925–2010, National Laboratory Animal Standardization Technical Committee). The current study was performed with license number of 20110611–01 approved by the Laboratory Animal Ethical Committee of China Agricultural University.

### Statistical Analysis

Statistical analyses were performed by using the Prism program (Prism GraphPad 7 software). Student’s *t*-test was performed for comparison between two groups; while one-way or two-way ANOVA was performed for analysis of multiple groups followed by Bonferroni’s or Tukey’s *post hoc* test. All cells experiments were performed three times independently, data are presented as mean ± SD. Significant differences were assigned as ^∗^*P* < 0.05, ^∗∗^*P* < 0.01, ^*⁣**^P < 0.001, and ^*⁣*⁣**^P < 0.0001, respectively.

## Results

### *In vitro* Cytotoxicity of Sodium Butyrate

**FIGURE 1:**
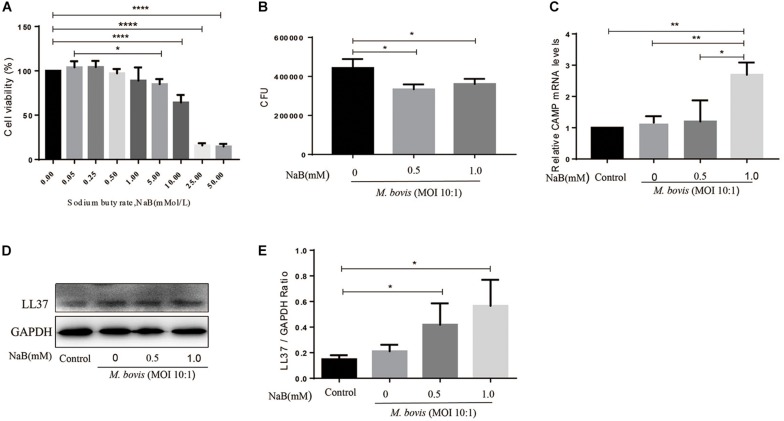
Sodium butyrate promotes the expression of antibacterial peptide LL37 and inhibits intracellular growth of *M. bovis* in THP-1 cells. **(A)** THP-1 cells were cultured in the presence of various concentrations of sodium butyrate (NaB) (0, 0.05, 0.25, 0.5, 1.0, 5.0, 10.0, 25.0, and 50.0 mM) for 24 h. Cell viability was determined by using MTS assay. THP-1 cells were treated with different concentration of NaB (0.5 and 1.0 mM) for 24 h followed by infection with *M. bovis* (MOI 10:1). **(B)** Colony-forming units (CFU) assay was performed for enumeration of viable *M. bovis* bacilli. The expression of LL37 was measured by **(C)** qRT-PCR and **(D)** western-bolt technique. **(E)** Relative band density of LL37 was calculated by using GAPDH as internal control. Data are representative of three independent experiments and values are expressed as mean ± SD. *P < 0.05; **P < 0.01; ****P < 0.0001.

In order to investigate the toxic effect of NaB on THP-1 cells, we used MTS assay. THP-1 cells were cultured in 96 wells culture plats with increasing concentrations of NaB (0, 0.05, 0.25, 0.5, 1.0, 5.0, 10.0, 25.0, and 50.0 mM) for 24 h in RPMI-5% FBS cell culture medium. As shown in Figure 1A, cell viability was not influenced by NaB at concentrations up to 1.0 mM. However, a concentration of NaB of 5.0 mM and above significantly affected the viability of THP-1 cells (Figure 1A). Based on these results, we selected a concentration of 0.5 and 1.0 mM for subsequent cell experiments.

### Sodium Butyrate Inhibits *M. bovis* Proliferation in THP-1 Cells

To investigate the effects of NaB on the intracellular survival of *M. bovis*, we infected THP-1 cells with *M. bovis* followed by treatment with NaB (0.5 and 1.0 mM) for 24 h. As shown in Figure 1B, we found a significant reduction in the total viable count of *M. bovis* in NaB (0.5 and 1.0 mM) treated cells compared to untreated controlled group (Figure 1B). These observations demonstrate that NaB augment macrophage-mediated killing of *M. bovis*.

### Sodium Butyrate Promotes the Expression of LL37 in THP-1 Cells Infected With *M. bovis*

As an innate immune effector molecules, LL37 is a human cathelicidin antimicrobial peptide encoded by *CAMP* gene, plays an important role in the control of infectious diseases (Vandamme et al., 2012). To evaluate the effect of NaB on the expression of LL37 in macrophages during *M. bovis* infection, therefore THP-1 cells were infected with *M. bovis* followed by treatment with NaB (0.5 and 1.0 mM) for 24 h. Total protein and mRNA samples were collected for quantification of LL37 level by western bolt and qRT-PCR assay. As shown in Figure 1, we found an increased expression of LL37 at both mRNA (Figure 1C) and protein (Figures 1D,E) level upon NaB treatment compared with untreated controlled cells post *M. bovis* infection. These results indicate that treatment of THP-1 cells with NaB during *M. bovis* infection promotes the expression of LL37 an antimicrobial peptide.

### Sodium Butyrate Inhibits the Expression of Histone Deacetylase in THP-1 Cells Infected With *M. bovis*

**FIGURE 2:**
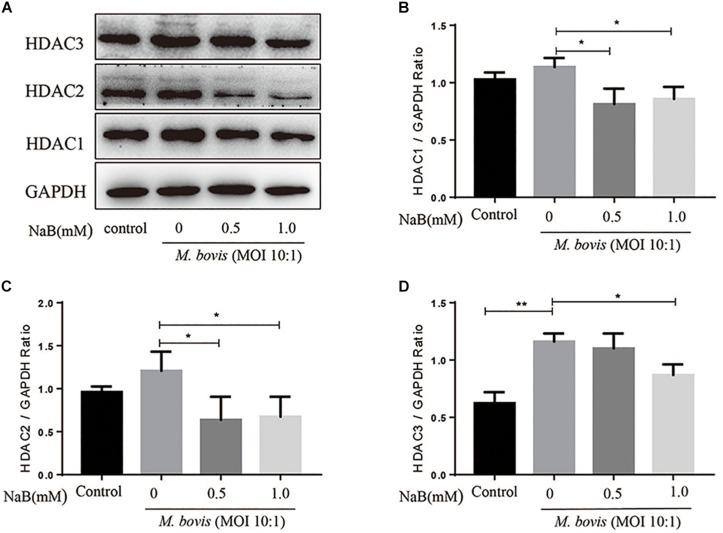
Sodium butyrate inhibits the expression of type I HDAC in THP-1 cells infected with *M. bovis*. THP-1 cells were treated with different concentration of NaB (0, 0.5, and 1.0 mM) for 24 h followed by infection with *M. bovis* (MOI 10:1). **(A)** The expression of HDAC1, HDAC2, and HDAC3 were determined by western bolt assay. The relative band density of **(B)** HDAC1, **(C)** HDAC2, and **(D)** HDAC3 were calculated by using GAPDH as internal control. Data are representative of three independent experiments and values are expressed as mean ± SD. *P < 0.05; **P < 0.01.

Histone deacetylase is an important regulator of gene transcription and plays a key role in a variety of immune responses. HDAC1, HDAC2, and HDAC3 belong to Class I HDAC, here we investigate whether NaB treatment affect the expression of Class I HDAC in macrophages infected with *M. bovis*. Therefore, we assessed the protein levels of HDAC1, HDAC2, and HDAC3 by WB assay in macrophages infected with *M. bovis* (MOI 10:1) in the presence or absence of NaB treatment. As shown in Figure 2, we observed a substantial increase in the protein levels of HDAC1, HDAC2, and HDAC3 in macrophages infected with *M. bovis*. In contrast, NaB (0.5 and 1.0 mM) treatment significantly reduced the expression of HDAC1 (Figure 2B), HDAC2 (Figure 2C), and HDAC3 (Figure 2D) in macrophages infected with *M. bovis* compared with untreated infected group. These results suggest that NaB potentially inhibits the expression of Class I HDAC in *M. bovis* infected THP-1 cells.

### Sodium Butyrate Inhibits the Activation of NF-κB Signaling Pathway in THP-1 Cells Infected With *M. bovis*

**FIGURE 3:**
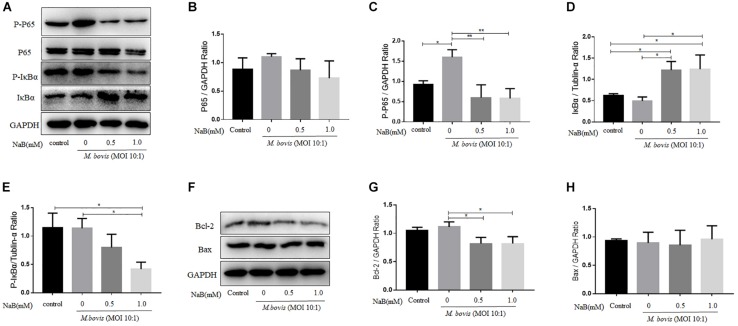
Sodium butyrate modulates NF-κB signaling pathway and Bcl-2 expression in THP-1 cells infected with *M. bovis*. THP-1 cells were treated with different concentration of NaB (0, 0.5 and 1.0 mM) for 24 h prior to infection with *M. bovis* (MOI 10:1). **(A)** The expression of P65, p-P65, IκBα, and P-IκBα were detected by western blot assay. The relative band density of **(B)** P65, **(C)** p-P65, **(D)** IκBα, and **(E)** P-IκBα were calculate by using GAPDH or Tublin-α as internal control. THP-1 cells were treated with different concentration of NaB (0, 0.5 and 1.0 mM) for 24 h prior to infection with *M. bovis* (MOI 10:1). **(F)** The expression of apoptosis-related proteins Bcl-2 and Bax were detected by western bolt assay. The relative band density ratio of **(G)** Bcl-2 and **(H)** Bax were calculated by using GAPDH as internal control. Data are representative of three independent experiments and values are expressed as mean ± SD. *P < 0.05; **P < 0.01.

Nuclear factor-κB signaling pathway plays an important role in the regulation of a large array of gene involved in different processes of the immune mechanism including inflammatory responses and apoptosis (Lu et al., 2013). To investigate the effect of NaB on the activation of NF-κB signaling pathway during *M. bovis* infection. We determined the expression of key component of NF-κB signaling cascades in infected THP-1 cells with *M. bovis* in the presence or absence of NaB treatment. As show in Figure 3A, the expression levels of p-P65 (Figure 3B) and IκBα (p-IκBα) (Figure 3C) were markedly increased in THP-1 cells infected with *M. bovis*. However, no significant difference was observed in the expression of P65, while a clear reduction in the expression level of IκBα (Figure 3D) was found during *M. bovis* infection. In contrast, NaB (0.5 and 1.0 mM/ml) significantly down-regulated the expression levels of p-P65 and p-IκBα, while up-regulated the expression of IκBα in *M. bovis* infected THP-1 cells (Figures 3C,E). The above results demonstrate that *M. bovis* induce the activation of NF-κB signaling pathway in infected THP-1 cells. However, NaB inhibits the activation of the NF-κB pathway triggered by *M. bovis* infection.

### Sodium Butyrate Modulates Apoptosis in THP-1 Cells Infected With *M. bovis*

It has been studied that apoptosis is a programed cell death process, which plays an important role in the clearance of intracellular bacteria including mycobacterium by host macrophages (Aberdein et al., 2013). In addition, NF-κB signaling pathway is an important modulator for the induction of apoptosis (Yin and Yu, 2018). In the early experiment, we found that NaB inhibited the activity of NF-κB signaling pathway. Next, we investigate the effect of NaB on the expression of apoptosis markers. Therefore, THP-1 cells were treated with NaB (0.5 and 1.0 mM) after infection with *M. bovis* (MOI 10:1) for 24 h. By using WB technique, we detected the expression of anti-apoptotic protein Bcl-2 and pro-apoptotic protein Bax. As shown in Figure 3F, we found that NaB treatment significantly reduced the expression of Bcl-2 (Figure 3G), while no effect was observed on the expression of Bax (Figure 3H) in *M. bovis* infected macrophages. Collectively, these results suggest that NaB modulates apoptosis during *M. bovis* infection.

### Sodium Butyrate Inhibits the Secretion of Cytokines in THP-1 Cells Infected With *M. bovis*

**FIGURE 4:**
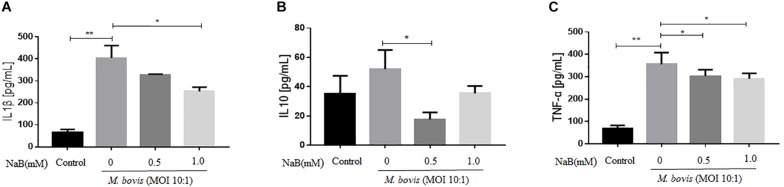
Sodium butyrate inhibits secretion of inflammatory cytokines in THP-1 cells infected with *M. bovis*. THP-1 cells were treated with different concentration of NaB (0, 0.5 and 1.0 mM) for 24 h prior to infection with *M. bovis* (MOI 10:1). The level of **(A)** IL-1β, **(B)** IL-10, and **(C)** TNF-α were detected by ELISA in the cell’s supernatant. Data are representative of three independent experiments and values are expressed as mean ± SD. *P < 0.05; **P < 0.01.

Although, NF-κB signaling pathway plays a key role in the secretion of cytokines, while in the early experiment we found that NaB attenuated the activation of NF-κB signaling pathway. Therefore, we examine the effect of NaB on the secretion of key cytokines by THP-1 cells infected with *M. bovis* after treatment with NaB. ELISA results revealed that *M. bovis* infection induced the secretion of IL-1β, IL-10, and TNF-α after 24 h of infection in THP-1 cells (Figures 4A–C). In contrast, NaB (0.5 and 1.0 mM) treatment significantly inhibited the production of IL-1β, IL-10, and TNF-α in THP-1 cells triggered by *M. bovis* infection (Figures 4A–C). Altogether, these findings suggest that NaB effectively subside the inflammatory reactions induced by *M. bovis* infection.

### Sodium Butyrate Treatment Enhances Resistance of Mice to *M. bovis* Infection

**FIGURE 5:**
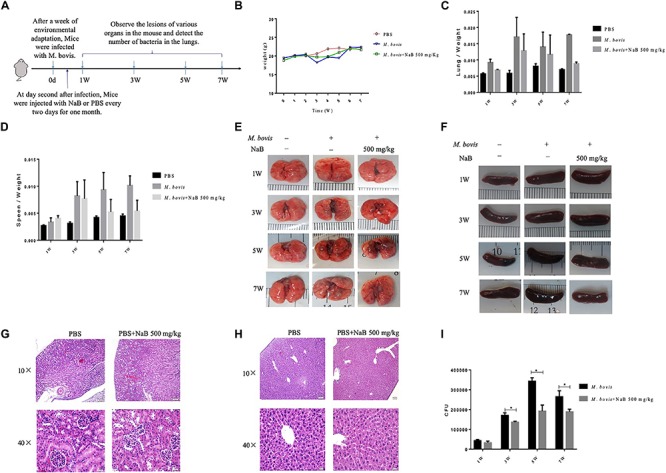
The effect of sodium butyrate on the pathogenesis of *M. bovis* infection in mice. **(A)** C57BL/6 mice were infected with 200 CFU of *M. bovis* via intranasal route followed by treatment with NaB (500 mg/kg) on every 2 days of interval via intraperitoneal injection (*n* = 6). **(B)** The effect of NaB treatment on total body weight of mice were determined at weekly interval post *M. bovis* infection. Organ coefficient of **(C)** lung and **(D)** spleen was determined post *M. bovis* infection upon treatment with NaB. Representative images for gross observation of **(E)** lung and **(F)** spleen of mice infected with *M. bovis* followed by treatment with NaB. Representative images of H&E stained sections of **(H)** liver and **(G)** kidney of mice were assessed for toxicity of NaB. **(I)** Colony-forming units (CFU) assay was performed for enumeration of viable *M. bovis* bacilli in the lung tissues of mice infected with *M. bovis* followed by treatment with NaB. Two-way ANOVA followed by Bonferroni’s multiple comparison test was performed for statistical analysis of data. *P < 0.05.

**FIGURE 6:**
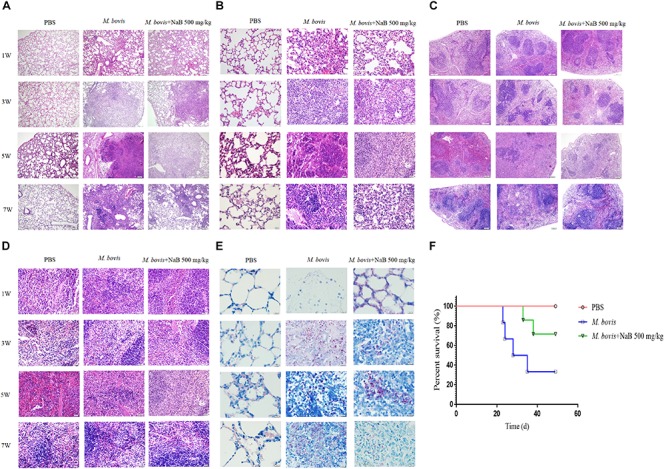
Histopathological study of lung and spleen tissues of mice infected with *M. bovis*. Representative images of H&E stained sections of lung tissues at **(A)** low power of magnification 10× and **(B)** high power of magnification 40× of mice infected with *M. bovis* followed by treatment with NaB. **(C)** Representative images of H&E stained sections of spleen tissues at low power of magnification 10× and **(D)** high power of magnification 40× of mice infected with *M. bovis* followed by treatment with NaB. **(E)** Representative images of Ziehl–Neelsen stained sections of lung tissues at 100× of mice infected with *M. bovis* followed by treatment with NaB. **(F)** Survival graph of mice infected with *M. bovis* followed by treatment with NaB. Scale bar: 50 μm **(A,C)**; scale bar; 20 μm **(B,D)**; scale bar; 10 μm **(E)**.

In cell experiments, we found that NaB potentially promoted the antibacterial ability of macrophages and inhibited the intracellular growth of *M. bovis*. Therefore, in order to determine whether NaB can prevent the pathogenesis of *M. bovis* in mice, we treated mice with PBS as a control and 500 mg/kg NaB as a treatment via i.p. route after 1 week of *M. bovis* infection. As shown in Figure 5A. We described a schematic presentation of mouse model of *M. bovis* infection and treatment with NaB and sample collection at various time periods post-infection. In addition, to determine the effect of NaB treatment on the pathogenesis of *M. bovis* in mice, we investigate the body weight of mice post-infection at various time points. Notably, we observed a significant loss in the total body weight of mice at 3rd and 5th week post-infection in untreated group compared with NaB treated group; however, no clear difference in the total body weight was observed at 7th week post-infection between treated and untreated mice (Figure 5B). Gross examination of lung tissues resulted that grayish white nodular lesions were appeared at 3rd week post infection, while clear and enlarged lesions were found in untreated mice compared with NaB treated mice at 5th and 7th week post-infection (Figure 5E). Similarly, spleen also showed lesions in the form of variable degree of swelling which was more pronounced in untreated mice compared with treated mice post *M. bovis* infection (Figure 5F). The organ coefficient of lung and spleen showed a clear increase in both lung and spleen weight in untreated infected group compared to NaB treated group at all times points post-infection except 1st week post-infection with *M. bovis* (Figures 5C,D). Histopathological examination was performed to investigate any toxic effect of NaB injection on mice infected with *M. bovis*. We found that microscopic observation of H&E stained tissue sections of liver and kidney showed no lesion or deformity in the normal architecture of kidney (Figure 5G) and liver (Figure 5H) of NaB treated mice compared with untreated groups post *M. bovis* infection. Furthermore, CFU results revealed a significant reduction in the total viable *M. bovis* bacilli in the lung tissues of NaB treated mice compared with untreated mice at 5th and 7th week post *M. bovis* infection (Figure 5I). Similarly, Ziehl–Neelsen stained lung sections showed reduced dissemination of *M. bovis* bacilli in NaB treated mice compared with untreated mice (Figure 6E). For detail study of lung’s lesions induced by *M. bovis* in infected mice after NaB treatment, we performed microscopy of H&E stained sections of lung. As shown in Figure 6, we found pulmonary epithelial hyperplasia and inflammatory cells infiltration was comparatively high in untreated groups than treated group at 3rd, 5th, and 7th week post-infection (Figure 6A). Under high magnification, we observed that the inflammatory cells in the lesion were mainly infiltrated by lymphocytes, neutrophils, and macrophages. Notably, the degree of lesions in the lung sections of mice treated with NaB was lower than untreated mice (Figure 6B). Similarly, an uneven distribution of white pulp and red pulp of the spleen was observed in H&E stained sections of spleen from untreated mice infected with *M. bovis.* In addition, an increased disturbance in the architecture of the spleen was observed in untreated mice compared with NaB treated mice pot *M. bovis* infection (Figures 6C,D). Furthermore, we found that NaB significantly improved the survival rate of mice, which was about 65% compared with untreated mice, recorded as 35% post *M. bovis* infection (Figure 6F). Collectively, the above results indicate that intraperitoneal injection of NaB reduces the pathogenesis and intracellular survival of *M. bovis* in infected mice.

## Discussion

As an intracellular bacterium, *M. bovis* is mainly phagocytosed by host macrophages. The interaction between *M. bovis* and macrophages is important to resolve or establish an infection (BoseDasgupta and Pieters, 2018). Early study reported that small molecules are key mediator of macrophage anti-mycobacterial activity for the elimination of intracellular *M. bovis* (Harbut et al., 2018). In addition, it has been studied that NaB promoted the expression of LL37 an important antibacterial peptide (Ochoa-Zarzosa et al., 2009). Furthermore, LL37 being an antimicrobial peptide that plays an important role in the control of mycobacterial infection mediated by regulation of protective host cell immune responses. In this study, we investigate the effect of NaB on macrophage activation during *M. bovis* infection. In addition, we also determine the effect of NaB on mouse model of *M. bovis* infection. Here, we found that NaB reduced the intracellular growth of *M. bovis* in THP-1 cells. Furthermore, our *in vivo* study showed that NaB treatment clearly minimized the severity of *M. bovis* infection in mice.

Emerging studies focus on the epigenetic modification of important gene expression by pathogens that affect the host immune responses (Dubovsky et al., 2010). In addition, epigenetic changes tend to regulate gene expression in a wider range associated with attenuation in the cell functions (Gomez-Diaz et al., 2012). Histone acetylation is one of post-translation modifications of histone, which plays an important role in the structural modification of chromosomes and regulation of gene expression. Intracellular pathogens such as *M. tuberculosis*, *Brucella*, *Leishmania* are the successor in manipulating host cell immune responses via Histone acetylation modification (Jeon et al., 2014). HDACs and HATs co-regulates the acetylation states of histones, and the epigenetic remodeling of histones in macrophages for the regulation of gene expression. In the present study, we found that NaB inhibited histone acetylation in THP-1 cells infected with *M. bovis*. Similarly, early study reported that NaB is known as a short-chain fatty acid type of HDACi that mainly affects the activity of Class I and Class II HDACs (Lorenz et al., 2011). In addition, we found a significant reduction in the expression of HDACs and viable count of *M. bovis* bacilli upon NaB treatment.

Previous research reported that HDAC participates in various intracellular immune responses, HDAC3 and HDAC2 participates in the acetylation of P65 to regulate the function of NF-κB (Rosemary et al., 2003). In addition, it has been reported that HDAC1 also play an essential role in the regulation of iNOS, IL-6, and IL-12B expression (Aneesh et al., 2015). Here, we found a crucial role of NaB on the regulation of HDAC1, HDAC2, HDAC3, and NF-κB pathway during *M. bovis* infection. Early study reported that the level of immune responses mediated by macrophages and the virulence factors of *M. bovis* determines the state of infection within the host (Huang et al., 2018). In the process of host-*M. bovis* interaction, various defense factors emerged to resist the development of infection by pathogens. Previous studies showed that host-pathogen interaction upregulate LL37 expression; however, the *M. bovis* induced subversion of cyclic AMP (cAMP) signaling inhibits LL37 expression (Gupta et al., 2017). Similarly, we demonstrated that *M. bovis* infection reduced the expression of LL37, while NaB treatment upregulated the expression of LL37 in THP-1 cells. NF-κB signaling pathway plays a fundamental role in the regulation of host cells inflammatory responses and apoptosis (Pando and Verma, 2000). In the present study, we found that NaB promoted the phosphorylation of P65, while inhibited the phosphorylation of IκBα. Previous study reported that *M. bovis* induced the induction of pro-inflammatory cytokines via activation of NF-κB signaling pathway in macrophages (Wang et al., 2016). However, recent study reported that NaB inhibited LPS-induced NF-κB signaling (Marinho et al., 2018).

Apoptosis is being a programed cell death process and also crucial to the host defense mechanism against intracellular pathogens. Furthermore, apoptosis is critical to expose pathogenic microorganisms to extracellular humoral immunity and prevent pathogenic microorganisms from utilizing host nutrition (Gao and Kwaik, 2000). In the current study, we observed that NaB induced apoptosis in *M. bovis* infected THP-1 cells via inhibiting the expression of anti-apoptotic marker protein Bcl-2. Our findings are in agreement with the previous investigation, reported that NaB induced apoptosis in human glioma cells (Sawa et al., 2001).

NaB, one of HDACi, affects different body functions including host innate immune defense mechanism. It is known that HDACi has been used to treat diseases such as tumors and epilepsy, but still need to explored the role of HDACi in the treatment of pathogenic microorganisms. Similarly, the study of Xiong et al. (2016) showed that NaB upregulated endogenous host defense peptides to enhance disease resistance in piglets. In the present study, we observed the effect of NaB on immune response of mice infected with *M. bovis*. Notably, we found that NaB treatment significantly reduced the intracellular growth of *M. bovis*, minimized the intensity of lesions in lung and spleen and prolonged the survival of mice post-infection with *M. bovis*. Similar to previous reports, we found that NaB has the potential to prevent the pathogenesis of *M. bovis* in mice.

## Conclusion

In conclusion, we explored that NaB enhanced host defense mechanism against *M. bovis* infection. We found that NaB not only increased the expression of LL37, but also promoted Histone acetylation by inhibiting type I HDAC. In addition, we also observed the anti-inflammatory and pro-apoptotic effect of NaB. The antibacterial ability of macrophages upon treatment with NaB depends on overexpression of LL37 and modulation of apoptosis during *M. bovis* infection. In addition, NaB suppressed the activation of the NF-κB signaling pathway that might a key target in the treatment of *M. bovis* infection. Interestingly, NaB treatment significantly reduced the severity of *M. bovis* infection in mice. Collectively, these observations illustrate that NaB could be used an adjunctive therapy for the treatment and prevention of *M. bovis* infection in animals and human beings.

## Data Availability Statement

The raw data supporting the conclusion of this article will be made available by the authors, without undue reservation, to any qualified researcher.

## Ethics Statement

All animal experiments were conducted under the protocols and procedures of the Chinese Regulations of Laboratory Animals—The Guidelines for the Care of Laboratory Animals (Ministry of Science and Technology of People’s Republic of China) and Laboratory Animal Requirements of Environment and Housing Facilities (GB 14925–2010, National Laboratory Animal Standardization Technical Committee). The current study was performed with license number of 20110611–01 approved by the Laboratory Animal Ethical Committee of China Agricultural University.

## Author Contributions

KZ wrote the manuscript. LX and XZ designed the experiments. KZ, TH, JW, ML, WW, XM, YL, and JY helped in performing WB, ELISA, qRT-PCR, and H&E staining. KZ, JW, YS, and ZL assisted in the animal experiments. TH helped in English correction before the final submission of the manuscript.

## Conflict of Interest

The authors declare that the research was conducted in the absence of any commercial or financial relationships that could be construed as a potential conflict of interest.
